# Quantifying performance on an outdoor agility drill using foot-mounted inertial measurement units

**DOI:** 10.1371/journal.pone.0188184

**Published:** 2017-11-16

**Authors:** Antonia M. Zaferiou, Lauro Ojeda, Stephen M. Cain, Rachel V. Vitali, Steven P. Davidson, Leia Stirling, Noel C. Perkins

**Affiliations:** 1 Department of Mechanical Engineering, University of Michigan, Ann Arbor, Michigan, United States of America; 2 Department of Orthopedic Surgery, Rush University Medical Center, Chicago, Illinois, United States of America; 3 Department of Aeronautics and Astronautics, Massachusetts Institute of Technology, Cambridge, Massachusetts, United States of America; University of Illinois at Urbana-Champaign, UNITED STATES

## Abstract

Running agility is required for many sports and other physical tasks that demand rapid changes in body direction. Quantifying agility skill remains a challenge because measuring rapid changes of direction and quantifying agility skill from those measurements are difficult to do in ways that replicate real task/game play situations. The objectives of this study were to define and to measure agility performance for a (five-cone) agility drill used within a military obstacle course using data harvested from two foot-mounted inertial measurement units (IMUs). Thirty-two recreational athletes ran an agility drill while wearing two IMUs secured to the tops of their athletic shoes. The recorded acceleration and angular rates yield estimates of the trajectories, velocities and accelerations of both feet as well as an estimate of the horizontal velocity of the body mass center. Four agility performance metrics were proposed and studied including: 1) agility drill time, 2) horizontal body speed, 3) foot trajectory turning radius, and 4) tangential body acceleration. Additionally, the average horizontal ground reaction during each footfall was estimated. We hypothesized that shorter agility drill performance time would be observed with small turning radii and large tangential acceleration ranges and body speeds. Kruskal-Wallis and mean rank post-hoc statistical analyses revealed that shorter agility drill performance times were observed with smaller turning radii and larger tangential acceleration ranges and body speeds, as hypothesized. Moreover, measurements revealed the strategies that distinguish high versus low performers. Relative to low performers, high performers used sharper turns, larger changes in body speed (larger tangential acceleration ranges), and shorter duration footfalls that generated larger horizontal ground reactions during the turn phases. Overall, this study advances the use of foot-mounted IMUs to quantify agility performance in contextually-relevant settings (e.g., field of play, training facilities, obstacle courses, etc.).

## Introduction

Running agility is required for many sports and other physical tasks that demand rapid changes of direction (e.g., agility drills during military training, basketball, soccer, lacrosse, football, rugby, tennis, etc.). Understandably, most studies of agility and cutting maneuvers confine experiments to the laboratory, where standard optical motion capture methods are employed for human motion analysis; see, for example, [1–5]. In laboratory settings, it is challenging to quantify agility skill because the experimental setup may not emulate realistic situations. However, body-worn inertial measurement units (IMUs) now enable human motion analysis in outdoor and other contextually-relevant settings (e.g., field of play, training facilities, obstacle courses, work environment); see, for example, [6–13]. The resulting measurements will likely increase the validity of conclusions for context-specific human performance, including agility performance as described herein.

Turning agility can be defined as the ease with which a body changes direction [14]. Changing direction requires simultaneously satisfying linear and angular momentum requirements to redirect the body in the new (desired) direction [15,16]. Agility has been studied using a range of turning-while-running tasks with agility performance commonly defined by the time to complete the agility task [14,17]. However, agility tasks often embed both turning and straightaway running subphases, and different strategies may be employed for each subphase that impact the recorded completion time. Exposing the strategies used while approaching and completing each subphase of an agility run will certainly improve our understanding of human agility [11]. However, doing so will also require a deeper understanding of the underlying body movements that cannot be inferred from completion times alone. One means to understand these movements is to measure the motion of major body segments using body-worn IMUs.

Wearable IMUs have been used previously to study turning agility in a variety of contexts. Trunk- or pelvis-mounted IMUs have been employed to evaluate running agility in a five-cone drill [11], turning-while-walking activities [6,18–21] and turning strategies in slalom skiing [22]. Mancini et al. [23] and El-Gohary et al. [24] used foot and torso mounted IMUs to learn that people with Parkinson’s disease tended to use more footfalls and smaller turn angles (larger turning radii) than healthy controls. Central to our study, McGinnis et al. [11] revealed body control techniques during the turn phases within a five-cone agility drill using acceleration and angular rate data harvested from a sacrum-mounted IMU. A cluster analysis showed that one group of participants accelerated at the apex of each turn with their pelvis aligned in the direction of travel significantly more than did the remainder of the participants. We further the scope of McGinnis et al. [11] by employing foot-mounted IMUs to estimate foot trajectories in order to identify performance strategies used during straightaway and turn subphases of the five-cone agility drill used during military training. Building upon McGinnis et al. [11], Mancini et al. [23], and El-Gohary et al. [24], we expect that high performers will use small turning radii (as opposed to the strategies used by people with Parkinson’s disease) and large tangential accelerations (linked to the positive tangential acceleration observed at the apex of the turn in a group of participants).

In this study, we analyze data from foot-mounted IMUs to (1) leverage opportunities for drift correction during footfalls and (2) evaluate the extent to which meaningful performance outcomes can be identified with only two sensors. We estimate the trajectory, velocity and acceleration of each foot using a Kalman filtering method that identifies and corrects for sensor drift errors. In particular, the filter exploits the instances when a foot is momentarily at rest during each footfall (i.e. zero velocity updates) to correct for velocity drift error, a strategy originally developed for tracking pedestrians indoors [25] and later extended to study gait [7,10].

The objectives of this study were to define and measure agility performance for a military (five-cone) agility drill using data harvested from two foot-mounted IMUs. We hypothesized that shorter agility drill performance times would be observed with small turning radii and large tangential acceleration ranges and body speeds. These hypotheses were tested by comparing trials performed by 32 participants completing an outdoor five-cone agility drill as described next. In addition to the hypotheses tested, we also explored how foot-mounted IMUs could estimate horizontal ground reactions.

## Materials and methods

### Participants and procedures

**Fig 1:**
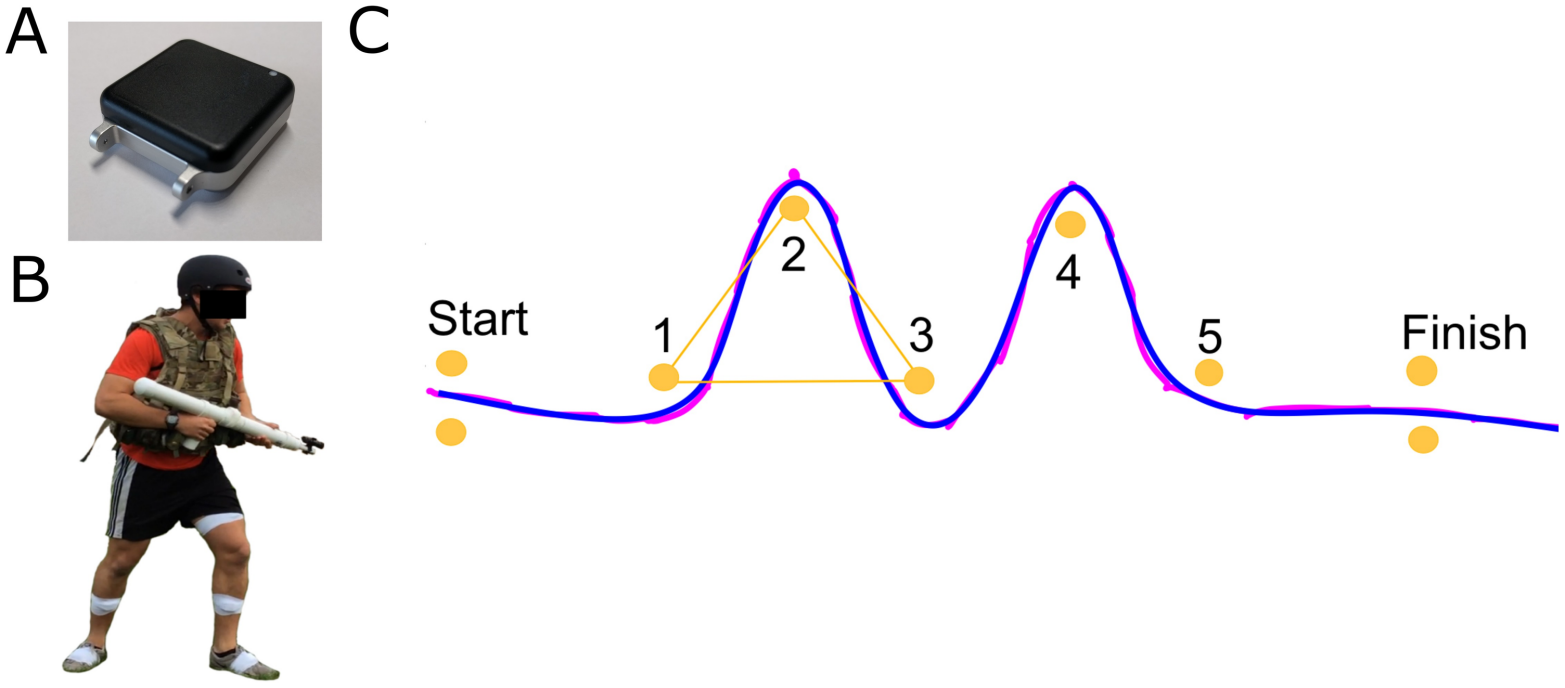
Participant and agility drill experimental setup and exemplar horizontal foot trajectory smoothing. (A) Photograph of the IMU selected for this study. (B) Participant wearing foot-mounted IMUs taped to the top of both shoes, additional body-worn sensors, and military accessories (because this study was part of an experiment designed to assess soldier performance of an outdoor obstacle course). (C) Agility drill setup with exemplar high performer (left) foot x-y trajectory: pre-smoothed (magenta) and and-post smoothed (blue). Cones (orange circles) are separated with 5m distance.

The University of Michigan Institutional Review Board approved this study. Thirty-two recreational athletes who were free of musculoskeletal injury (17M, 15F, mean (SD) age: 20.1 (2.1) years, height: 1.75 (.13) m, mass: 71.3 (13.7) kg) volunteered to participate and provided written informed consent in accordance with the University of Michigan Institutional Review Board. The individual in this manuscript (Fig 1B) has given written informed consent (as outlined in PLOS consent form) to publish these case details. Participants completed an outdoor obstacle course including a five-cone agility drill (Fig 1C). The agility drill was comprised of start and finish gates, and five cones that created a series of five turns: 60° turn left, 120° turn right, 120° turn left, 120° turn right, and 60° turn left with 5m between cones (Fig 1C). They were asked to complete the agility drill as quickly as possible, while circumventing each cone (without stepping over cones). Participants were given an opportunity to practice the agility drill following the prompt: “please practice as long as you want to practice” before the IMUs were affixed to their body segments. Participants were not provided with training on strategy for accomplishing the obstacle, allowing them to use strategies that were natural to them (from any prior experience/practice in their athletic history). Participants using self-selected agility drill strategies enabled identification of a variety of feasible navigation strategies and a wide spread of bottom-line performance, as measured by agility drill duration. During data collection, subjects performed the agility run between one and four times (depending on their random enrollment into participant groups). This study’s experimental protocol was a portion of a larger study designed to assess soldier performance (via whole body movement analyses) during an outdoor obstacle course that included the agility run obstacle. Therefore, some participants were chosen to repeat the agility run, whereas, others were selected to repeat other obstacles in the course.

### Instrumentation

Participants wore a pair of wireless IMUs (128Hz, Opal V1, APDM, Portland, OR) taped to the top of athletic shoes (Fig 1A and 1B). The IMUs utilized in this experiment were selected because they automatically synchronize up to 24 sensors, provide raw data to the researchers, have a long battery life (12 hrs), and ample internal storage (8 Gb). These sensors were used on “data logging mode” to ensure lossless data measurement via their internal storage system vs. streaming data. Each IMU contains a 3-axis accelerometer (±6 g range and 14-bits resolution), 3-axis gyroscope (±2,000 dps range and 14-bits resolution), and 3-axis magnetometer (±6 Gauss range and 12-bits resolution). Further technical specifications are available from the IMU manufacturer. Each IMU was packaged in a 48.4 x 36.1 x 13.4 mm plastic casing, weighing <22 g, that was secured to a fabric band, similar to the packaging of a smart watch (Fig 1A).

The IMUs were secured on the shoes by the same experienced researcher and tightly attached to the shoe top (laces) using athletic tape. Participants carried mock-rifles (3.4kg) for the purpose of understanding performance in this military obstacle course, including performance in the agility run (Fig 1B).

### Data reduction

Raw data from the 3-axis accelerometer and 3-axis angular rate gyroscope embedded in the IMUs were processed using custom algorithms. Inertial navigation equations were used to compute the attitude angles of the sensor axes relative to a field-fixed frame of reference as described by Savage [26] and a custom Kalman filter was used to correct for tilt errors using gravity as a reference [7,25]. The stationary times during foot/ground contact were detected using thresholds defining minimum acceleration (1 m/s) and angular rate (30°/s). A wavelet analysis was used to establish the beginning and end of each foot/ground contact period [27]. The estimated velocity of each foot was corrected for drift error by demanding that the foot velocity remain zero at the stationary time of each foot/ground contact period. Subsequent integration of the drift-corrected foot velocity yields the foot trajectory (i.e, x-y foot location vs. time) [25]. This trajectory was smoothed using the Matlab^™^ cubic spline function (threshold input p = 1e-6, Mathworks, Natick, MA), with an example smoothed trajectory shown in Fig 1C.

**Fig 2:**
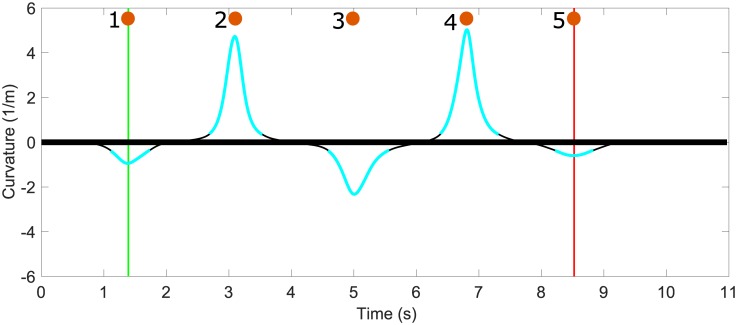
Exemplar average horizontal foot trajectory curvature. Average foot path curvature as a function of time for an exemplar high performer. Agility drill time is the time that elapses between a performer passing cone 1 and passing cone 5. The approximate time that the performer circumvented each cone is represented with numbered orange circles.

Each foot’s smoothed trajectory was used to automatically detect the turn and straightaway phases of this agility drill. These phases are immediately apparent in Fig 2, which illustrates the instantaneous curvature of the foot trajectory (average of both feet) that distinguishes the turn phases (blue segments) from the straightaway phases (black segments). In particular, turn phases were defined as times when the (average) curvature of the foot trajectory exceeded ± 0.37 (1/m); refer to Fig 2. The agility drill time, and the other IMU-derived performance metrics introduced below, were assessed between the time that the participant crossed the first and fifth cones (Fig 2). Doing so focused attention on the fraction of this drill where changing direction (i.e. agility skill) is the primary objective for the performer. The times when a performer passed a cone were deduced from the instantaneous curvature of the foot paths (Fig 2) noting that the curvature achieves an extremum value (either positive or negative) at a cone (Fig 2). The agility drill time is defined as the time of extreme curvature while circumventing the first cone until the time of extreme curvature while circumventing the fifth cone. Furthermore, this agility drill time was chosen as a measure of agility performance that is immune to participants’ interpretation of how to run through the finish gate (i.e., stop precisely on a designated mark 5 m beyond the finish gate, or to overshoot and then return to the mark). The agility drill time so defined is highly correlated with the time between the start and finish gates as measured with a stopwatch (R^2^>0.7).

**Fig 3:**
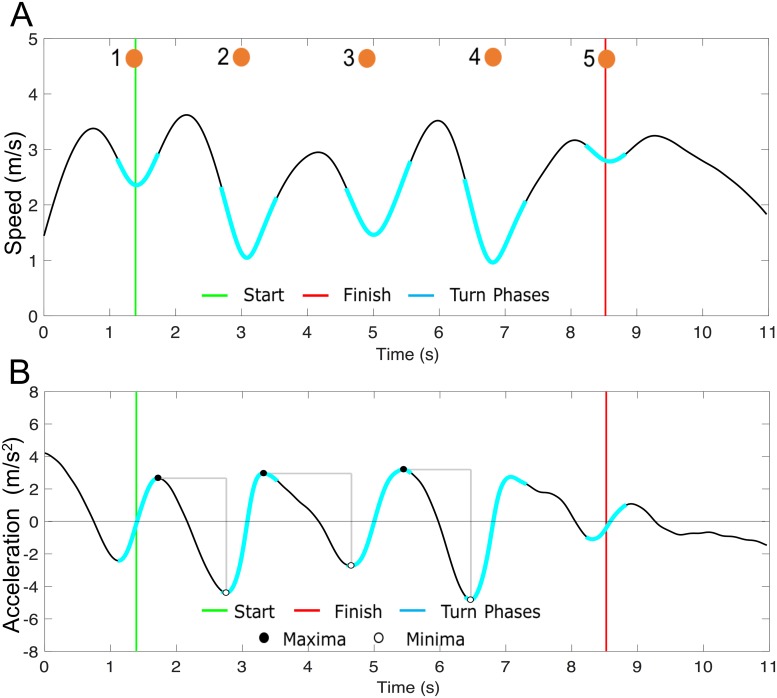
Exemplar body speed and tangential acceleration estimates. **(A)** Body speed and **(B)** tangential acceleration estimates vs. time for an exemplar high performer. The approximate time that the performer circumvented each cone is represented with numbered orange circles. The tangential acceleration range is represented by the vertical gray bars between successive cones.

Four agility performance metrics are proposed including: 1) agility drill time (defined above), 2) horizontal body speed, 3) turning radius of the foot trajectory, and 4) tangential body acceleration range. The resultant horizontal body speed is approximated as the average horizontal speed of both feet, assuming the body center of mass horizontal speed remains between that of the feet during running (Fig 3A) [28]. The turning radius follows from the reciprocal of the average curvature as a function of time (Fig 2). The tangential body acceleration range between a pair of cones was calculated by differentiating the body speed (Fig 3B). The aforementioned performance metrics were averaged during the middle three (120°) turn phases, where the greatest agility skill is required.

Additionally, the average horizontal ground reaction during each footfall was estimated using the linear impulse-momentum relationship (i.e., average horizontal ground reaction equals change in horizontal linear momentum of mass center divided by the duration of ground contact) and was normalized by body mass to compare across participants. We approximated the velocity of the performer’s mass center by the average velocity of the performer’s feet. In order to align the velocity vectors from each foot, the horizontal (x-y) trajectories from each foot were rotated (about the z-axis) such that the start and end positions of each foot were aligned with a constant y-value (matching the agility drill course geometry). The average horizontal ground reaction at each foot was further resolved into tangential and normal components relative to the horizontal foot trajectory of each foot (e.g., the tangential component defined by the dot product of the horizontal ground reaction and a unit vector in the direction of the foot velocity during single-leg support). The ground contact time for each footfall during a turn phase was summed to define a “cumulative footfall duration”. An additional turn phase metric, the “force-generation metric” was calculated as the average horizontal ground reaction during the turn phases divided by the cumulative footfall duration. This force-generation metric is therefore large whenever a participant generates a large horizontal ground reaction in a short period of ground contact time during the turn phases.

### Statistical analysis

Agility drill performance was divided into groups of “*high”*, *“mid”*, and *“low” performance* based on sorting all trials by agility drill time into three equal-sized groups. Nineteen trials were included per performance group due to multiple trials performed by the majority of subjects. In some cases, trials of the same performer spanned two performance groups (six participants had trials in both high and mid performance groups and two participants had trials in both mid and low performance groups; no participant had trials spanning all three performance groups). In order to distinguish the techniques that were used by the performance groups, non-parametric rank-based statistical methods were used (as the data did not meet the assumption for a parametric linear model and predictive models were not the outcome of interest). For each dependent variable, a Kruskal-Wallis omnibus test was performed to compare across performance groups as defined by agility drill time. When this test was significant, post-hoc comparisons were performed using Mean Rank tests (including a Bonferroni critical value correction for multiple comparisons) [29]. This statistical approach assumes independence between trials, both across-subject and within-subject: each trial is treated as a stand-alone observation, regardless of which participant performed the trial or the sequence of trial performance when the agility drill was repeated by a single participant.

## Results and discussion

### Results

**Fig 4:**
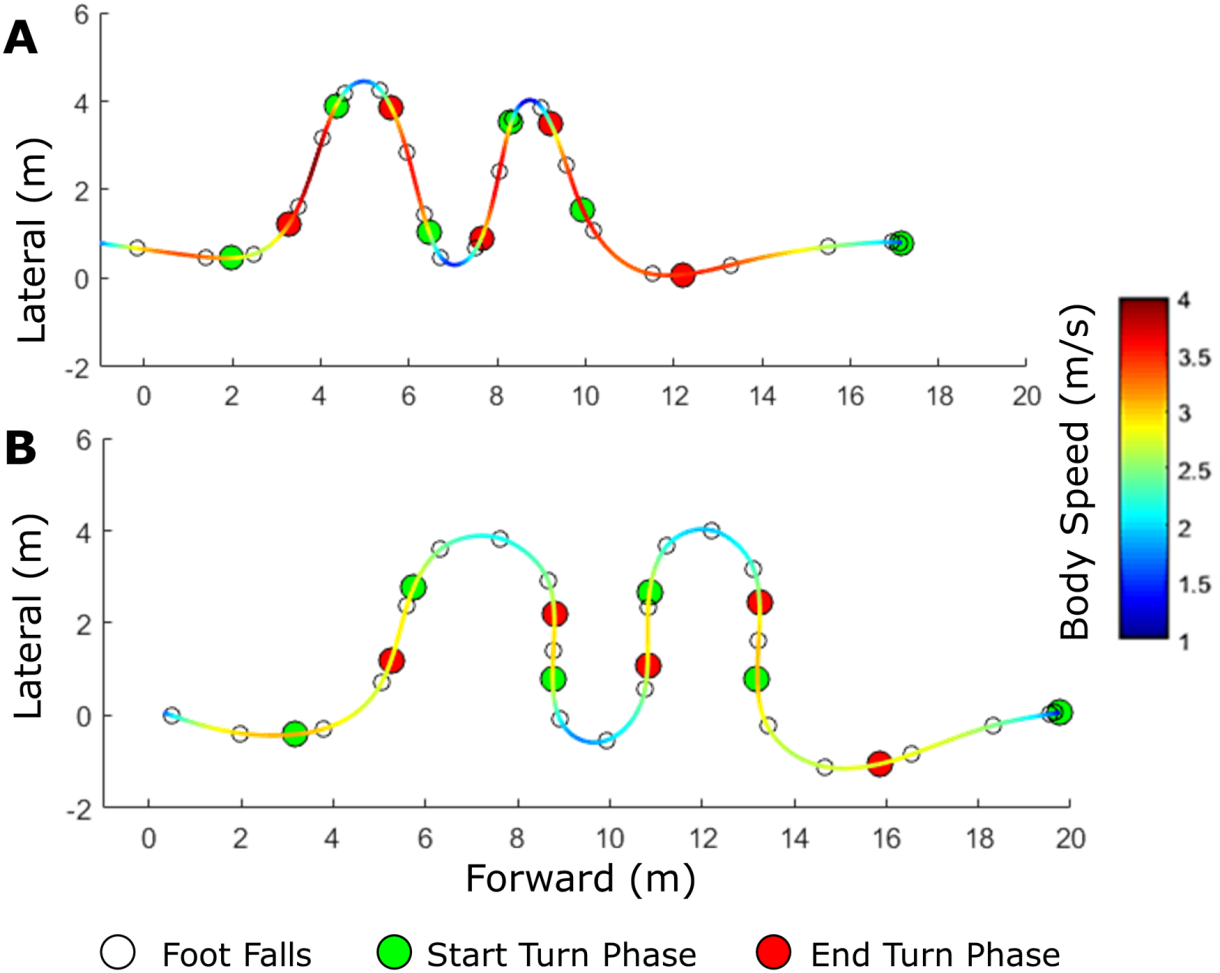
Exemplar right foot horizontal trajectories with body speed. Exemplar right foot horizontal trajectories with body speed (color scale) for a **(A)** high performer and a **(B)** low performer.

**Fig 5:**
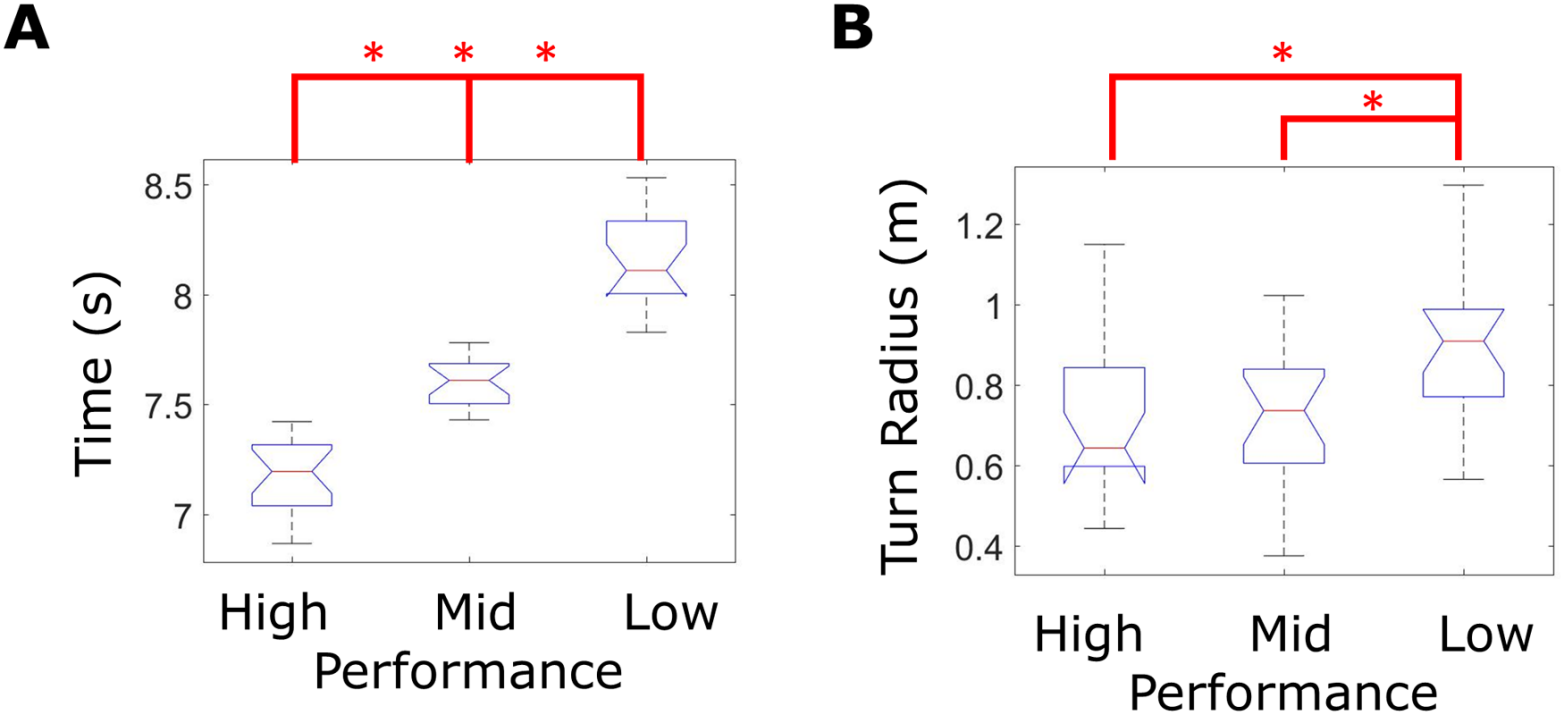
Agility drill time and average turn radius performance metric grouped by agility drill time. Boxplots of **(A)** agility drill time and **(B)** average turn radius for each trial evenly grouped by sorting agility drill time as the performance outcome. Asterisks indicate statistically significant differences between performance groups via post-hoc mean rank test (Table 1).

As expected, shorter agility drill time was observed with smaller turning radii (Figs 4 and 5, Table 1). Fig 4 displays exemplar x-y right foot trajectories and body speed for a high-performance trial versus a low-performance trial for reference throughout the results and discussion sections. Fig 5 displays boxplots for (A) agility drill time and (B) turn radius for the high, mid, and low performance groups. Agility drill time was statistically different between groups (omnibus p<0.001, Fig 5A, Table 1) defined by the following agility drill time ranges: high performance (6.9–7.4s), mid performance (7.4–7.8s), and low performance (7.8s-8.5s). Average turn radius used during low performance trials was significantly greater than that used during either high or mid performance trials (omnibus p = 0.003, Fig 5B, Table 1).

**Table 1:** Summary of statistical results.

Metric	Kruskal-Wallis		Post-hoc Mean Rank Test		
	*X*^2^	P-value	High vs. Low	High vs. Mid	Low vs. Mid
Agility Drill Time	49.80	**< .001**	**< .001** **[-50.6, -25.4]**	.**001** **[-31.6, -6.4]**	.**001** **[-31.9, -6.4]**
Turn Radius	11.39	.**003**	.**006** **[-29.6, -3.8]**	1.0 [-14.9, 10.8]	.**02** **[-27.5, -1.7]**
Tangential Acceleration Range	26.44	**< .001**	**< .001** **[13.6, 38.8]**	.58 [-7.3, 17.9]	**< .001** **[8.2, 33.5]**
Body speed	21.47	**< .001**	**< .001** **[12.3, 37.6]**	.**046** **[0.2, 25.4]**	.06 [-.5,24.8]
**Turn phase ground reaction-based performance metrics**					
Horizontal ground reaction magnitude	30.0	**< .001**	**< .001** **[16.0, 41.3]**	.283 [-4.5, 20.8]	**< .001** **[7.8, 33.1]**
Percent horizontal ground reaction normal to trajectory	9.48	.**009**	.**007** **[-28.9, -3.7]**	.517 [-18.5, 6.7]	.126 [-23.1, 2.1]
Cumulative footfall duration	21.49	**< .001**	**< .001** **[-36.0, -10.8]**	.720 [-16.7, 8.4]	.**001** **[-31.9, -6.6]**
“Force generation” metric	34.07	**< .001**	**< .001** **[17.7, 42.9]**	.284 [-4.5, 20.8]	**< .001** **[9.6, 34.8]**

Omnibus Kruskal-Wallis yielded a chi-square (*X*^2^) and p-value for each test. Post-hoc mean rank tests yielded a p-value and confidence interval (in brackets) for each test that compared between two performance groups. Bold font represents statistically-significant results when tested at α = 0.05 level.

**Fig 6:**
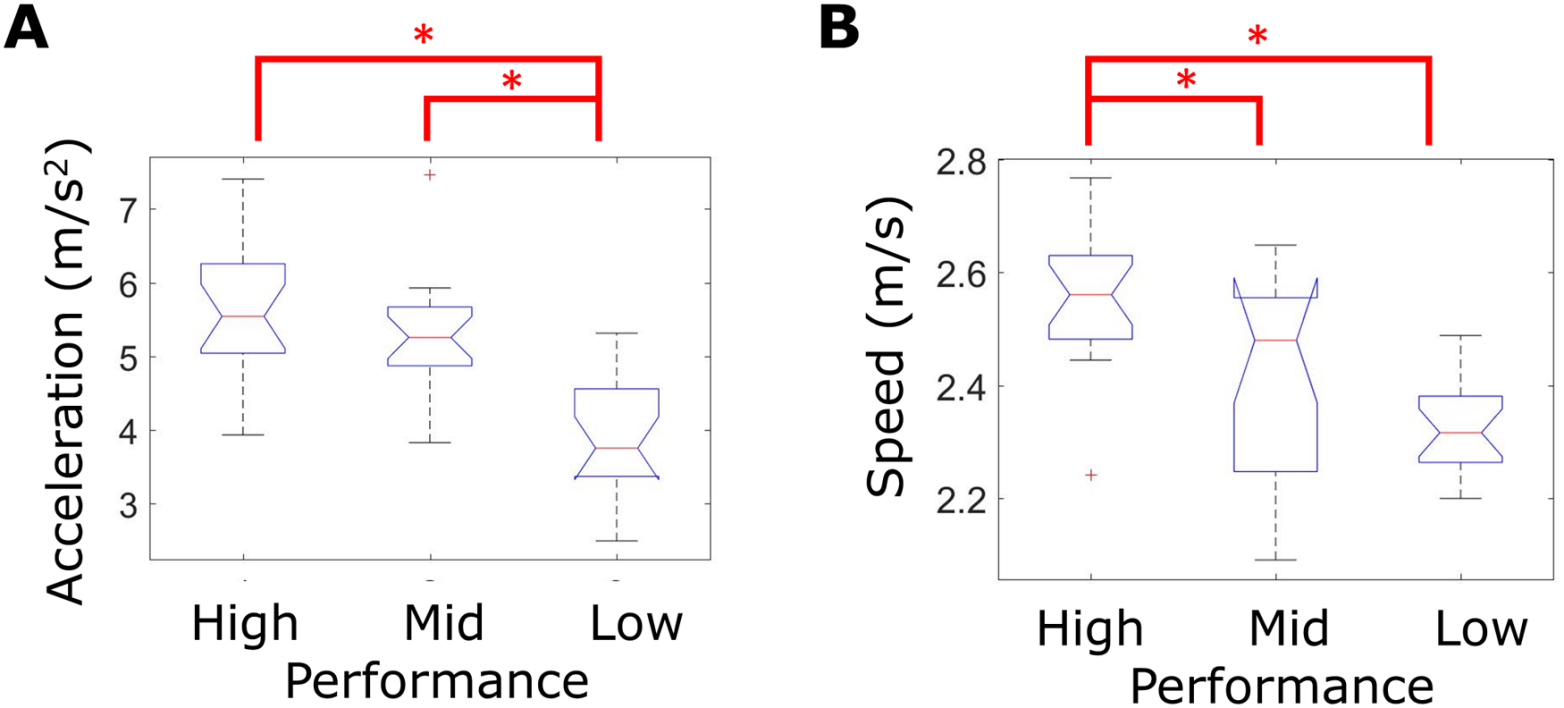
Tangential acceleration range and body speed performance metrics grouped by agility drill time. Boxplots of average estimated **(A)** tangential acceleration range and **(B)** body speed for each trial evenly grouped by sorting agility drill time as the performance outcome. Asterisks indicate statistically significant differences between performance groups via post-hoc mean rank test (Table 1).

As expected, shorter agility drill times were observed with larger average tangential acceleration ranges and average body speeds. Fig 6 displays boxplots of average (A) tangential acceleration range and (B) body speed for high, mid, and low performance groups (distinguished by agility drill time). The average tangential body acceleration range used during low performance trials was significantly smaller than that used during either mid or high performance trials (omnibus p<0.001, Fig 5A, Table 1). The average body speed estimate during high performance trials was significantly greater than that used during either mid or low performance trials (omnibus p<0.001, Fig 6B, Table 1).

**Fig 7:**
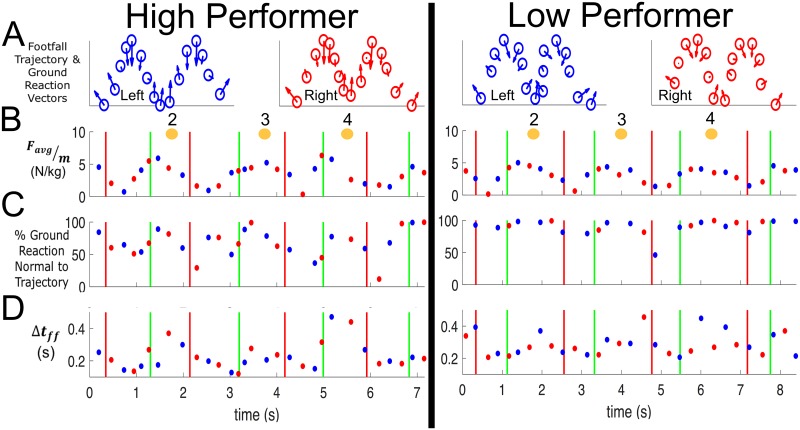
Exemplar ground reaction-based performance metrics. Exemplar (**A**) left and right foot trajectories with vectors showing the direction of the average horizontal ground reaction per footfall between cones 2 and 4, **(B)** average horizontal ground reaction (body mass normalized) during each footfall, **(C)** percent average horizontal ground reaction (body mass normalized) normal to the trajectory, and **(D)** ground contact time for each footfall. Data for right (red) and left (blue) footfalls are shown for the same high performer (left) and low performer (right) trials in Fig 4. Black boxes encapsulate footfall data points during turn phases. The numbered orange circles denote the approximate time when the performer circumvented each cone.

**Fig 8:**
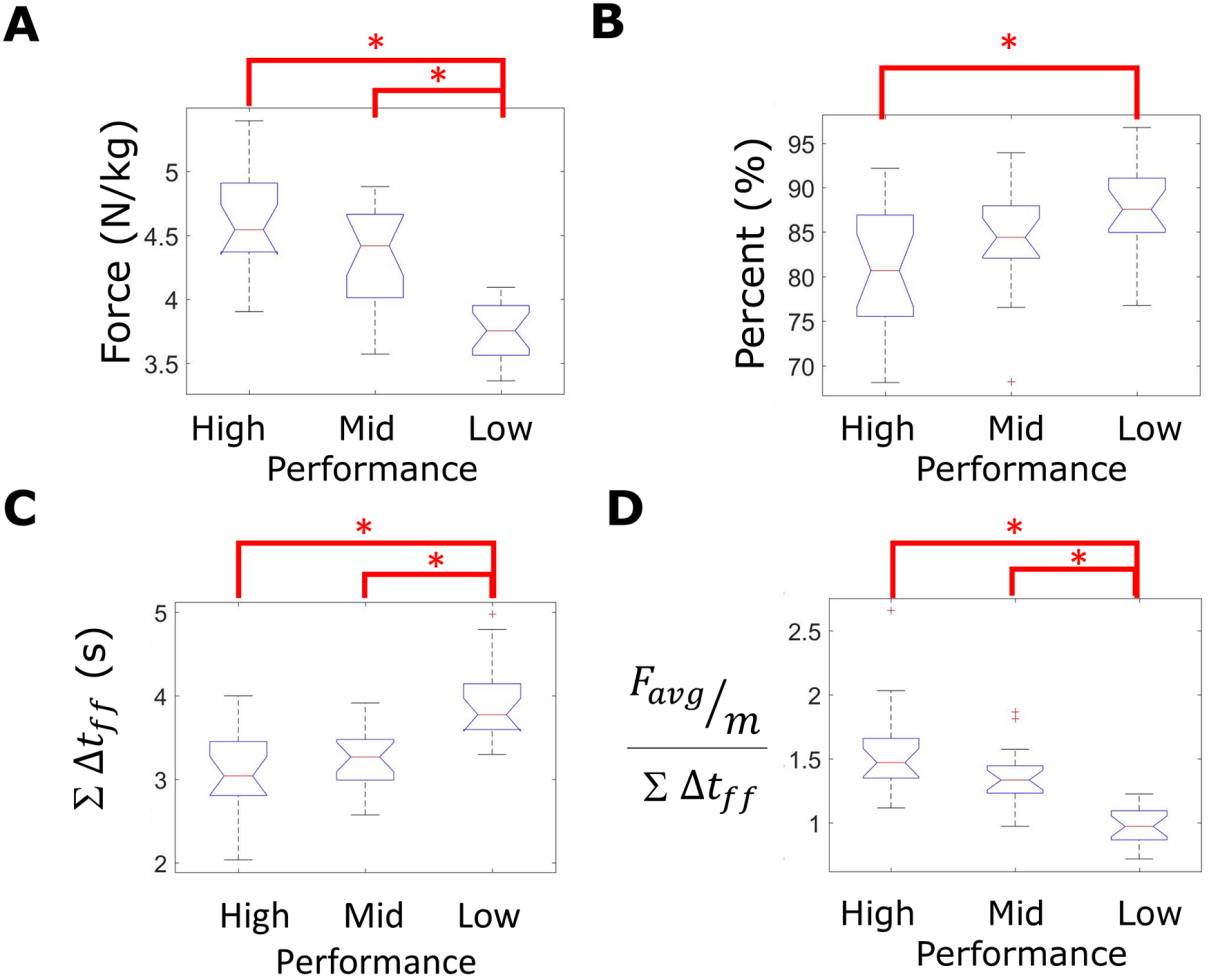
Turn phase ground reaction-based performance metrics grouped by agility drill time. Boxplots of ground reaction-based performance metrics for each trial evenly grouped by sorting agility drill time as the performance outcome. The following metrics are displayed, calculated during turn phases: **(A)** Average horizontal ground reaction magnitude (body mass normalized), **(B)** average percent horizontal ground reaction normal to the foot trajectory, **(C)** cumulative footfall duration, and **(D)** “force generation” metric. Asterisks indicate statistically significant differences between performance groups via post-hoc mean rank test (Table 1).

In addition to testing the hypotheses, results also highlight the influence of the estimated horizontal ground reaction on agility drill time. Fig 7 displays exemplar (A) average horizontal ground reaction vector, (B) average horizontal ground reaction magnitude, (C) the percentage of this ground reaction that is normal to the foot trajectory, and (D) the footfall duration. Results for the exemplar high performer (left) are distinguished from those of the exemplar low performer (right). Fig 8 displays the boxplots of horizontal ground reaction metrics for high, mid, and low performance groups (distinguished by agility drill time). Low performance trials generated significantly lower average horizontal ground reactions per footfall during the turn phases than did mid and high performance trials (omnibus p<0.001, Figs 7B and 8A, Table 1). Relative to low performance trials, the high performance trials exhibited significantly less ground reaction directed normal to the path trajectory (omnibus p = 0.009, Figs 7C and 8B, Table 1). Low performance trials exhibited significantly greater cumulative footfall duration during the turn phases than either mid or high performance trials (omnibus p<0.001, Figs 7D and 8C, Table 1). Finally, low performance trials exhibited significantly smaller force-generation metrics than did either mid or high performance trials (omnibus p<0.001, Figs 7B, 7D and 8D, Table 1).

### Discussion

This study elucidates distinct strategies used by high and low performers in this five-cone agility drill, as distinguished by agility drill time. As hypothesized, shorter agility drill performance times were observed with smaller turning radii, larger tangential acceleration ranges, and larger body speeds using Kruskal-Wallis and mean rank post-hoc statistical analyses. The exemplar data illustrated in Fig 4 clearly reveals these strategies. High performers treated the agility drill as a series of brief sharp turns between straightaway sprints characterized by large changes in body speed. In contrast, low performers used wide turn radii and maintained medium body speed throughout. High performers essentially “dumped” speed while entering a turn to negotiate the turn with small turning radii (high curvature). This strategy requires large deceleration while approaching a turn followed by large acceleration while leaving a turn, manifesting in large changes in tangential acceleration between turns and straightaways. In contrast, low performers tended to maintain medium body speed throughout the course (and modest changes in tangential acceleration) by using more steps and wider turning radii during turn phases. These results complement those of McGinnis et al. [11] because agility drill performance was distinguished by differences in 1) tangential pelvis acceleration at the apex of each turn, which is linked to this study’s finding regarding differences in tangential acceleration range, and 2) the alignment of the pelvis with the direction of travel, which is linked to this study’s finding that high performance trials used a series of straightaway sprints vs. continuous turns).

The trade-off between body speed and curvature observed in this study of running agility is also observed in the turns executed by slalom skiers [30], the turns performed by competition horses [31], and in the curved hand trajectories produced by coordinated movements of the upper limbs [32]. These findings regarding turning radius versus speed also link to the finding that fall-risk clinical populations tend to use “shuffle turn” strategies with larger turning radii and more footfalls [23,24,33,34].

Rapid changes in direction, signaled by short duration turn phases, require the rapid generation of linear and angular impulses from ground reactions. These impulses accelerate the center of mass of the body in a new (desired) direction and also re-orient the body towards that direction [15,16,35]. Compared to lower performers, higher performers in our study exhibited shorter duration ground contacts and generated larger horizontal ground reactions that also had larger components tangent to (less normal to) the turn trajectories. In contrast, lower performers exhibited longer duration and smaller horizontal ground reactions that remained largely normal to the turn trajectories. As a result, lower performers negotiated wider and longer turns, but at more uniform (medium) speed.

### Study limitations

By exploiting the use of body-worn IMUs, this study exposes agility performance in a natural context (i.e., outside of a laboratory), namely within (a portion of) an outdoor obstacle course. While this approach may increase the validity of the findings for field-based athletic and/or soldier performance, the experimental conditions were obviously less controlled than those in laboratory settings. In particular, there was no control of the length or moisture of the grass, the presence or absence of occasional leaves, slight irregularities or slopes in the terrain, all of which may have affected how participants planned and executed their runs (i.e., low friction, leaves or downward slope may increase slip risk). We also introduce a number of performance metrics based on approximating the trajectory, velocity and acceleration of a participant’s mass center as the average of those computed for the left and right feet (thereby ignoring the added components due to body lean). The same approximations underlie our estimates of the horizontal ground reactions. In addition, the trajectories of each foot are themselves estimated results employing inertial navigation algorithms. In particular, they rely on identifying when the foot is momentarily at rest to provide a “zero velocity update” for the subsequent integration time interval. This procedure can be challenged by temporary sensor saturation during footfalls, though that was rare due to the considerable compliance of the grass terrain. This study was executed within the context of an experiment focused on learning how a military obstacle course is performed. Therefore, (1) participants wore helmets and carried mock-rifles and (2) participants performed other obstacle course tasks before and after the agility drill. A within-subject pilot study of participants did not reveal significant differences (1) in agility drill time if the participant carried the rifle or not, (2) nor in agility drill time vs. agility trial order in participants who repeated the agility drill. However, using military accessories or performing other obstacle tasks could have altered performance in ways that were not detected statistically in the current and pilot studies. While the participants were given break time between obstacle course tasks, fatigue could have affected performance differently within-subject or across-subject. Regardless of potential fatigue-effects, this study’s statistical design enabled detection of a wide-variety of performance strategies and outcomes (each trial was assumed to be independent from participant or trial sequence). Finally, the participants of this study were recreational athletes who had different agility training backgrounds and were not trained during the study, which implies that certain cutting maneuvers could have been physiologically unavailable or unknown to certain performers. Within this cohort, an analysis of participant athletic background (i.e., rugby, triathlon, etc.) yielded no significant differences vs. agility drill time. However, in the future, it would be interesting to learn how amount of prior agility drill practice and specific agility training programs relate to performance.

## Conclusions

This study leverages the advantages of using two foot-mounted IMUs to study performance on an outdoor (five-cone) agility drill. The acceleration and angular velocity data harvested from the two IMUs were used to estimate foot (and mass center) trajectory, velocity and acceleration as well as horizontal ground reactions during foot-ground contact. These estimates reveal the following major distinctions between high and low-performing participants, with performance distinguished by the time to complete the agility run. Relative to low performance, high performance is characterized by sharper turns, larger changes in body speed (i.e., larger tangential acceleration range), and shorter duration footfalls that also generate larger, horizontal ground reactions during the turn phases. This study may motivate future extensions on running agility that incorporate additional IMUs to expose whole-body movement and body segment control and phasing. For instance, torso-mounted IMUs may reveal that added body lean during the turn phases enables even smaller turn radii for high performers. Furthermore, data from shank- and thigh-mounted IMUs, as in [36], may reveal non-hinge like knee behavior during the turn phases as found in other turning activities [37,38].

## Supporting information

S1 Table Results data table. Data for each agility run trial are included in this table.
(XLSX)

## References

[1] Havens KL, Sigward SM. Whole body mechanics differ among running and cutting maneuvers in skilled athletes. Gait Posture. 2015;42: 240–245. doi: 10.1016/j.gaitpost.2014.07.022 25149902 10.1016/j.gaitpost.2014.07.022

[2] Jindrich DL, Qiao M. Maneuvers during legged locomotion. Chaos An Interdiscip J Nonlinear Sci. 2009;19: 26105 doi: 10.1063/1.3143031 19566265 10.1063/1.3143031

[3] Suzuki Y, Ae M, Takenaka S, Fujii N. Comparison of support leg kinetics between side-step and cross-step cutting techniques. Sports Biomech. 2014;13: 144–53. doi: 10.1080/14763141.2014.910264 25122999 10.1080/14763141.2014.910264

[4] Besier TF, Lloyd DG, Cochrane JL, Ackland TR. External loading of the knee joint during cutting maneuvers. Med Sci Sport Exerc. 2001;1: 1168–1175. doi: 10.1097/00005768-200107000-00014 10.1097/00005768-200107000-00014 11445764

[5] Miranda DL, Rainbow MJ, Crisco JJ, Fleming BC. Kinematic differences between optical motion capture and biplanar videoradiography during a jump-cut maneuver. J Biomech. Elsevier; 2013;46: 567–573. doi: 10.1016/j.jbiomech.2012.09.023 23084785 10.1016/j.jbiomech.2012.09.023 PMC3551998

[6] Beyea J, McGibbon CA, Sexton A, Noble J, O’Connell C. Convergent validity of a wearable sensor system for measuring sub-task performance during the timed up-and-go Test. Sensors. 2017;17: 934 doi: 10.3390/s17040934 28441748 10.3390/s17040934 PMC5426930

[7] Rebula JR, Ojeda L V, Adamczyk PG, Kuo AD. Measurement of foot placement and its variability with inertial sensors. Gait Posture. 2013;38: 974–80. doi: 10.1016/j.gaitpost.2013.05.012 23810335 10.1016/j.gaitpost.2013.05.012 PMC4284057

[8] Peruzzi A, Della Croce U, Cereatti A. Estimation of stride length in level walking using an inertial measurement unit attached to the foot: A validation of the zero velocity assumption during stance. J Biomech. 2011;44: 1991–1994. doi: 10.1016/j.jbiomech.2011.04.035 21601860 10.1016/j.jbiomech.2011.04.035

[9] Seel T, Schauer T, Raisch J. Joint axis and position estimation from inertial measurement data by exploiting kinematic constraints. 2012 IEEE Int Conf Control Appl. Ieee; 2012; 45–49. http://ieeexplore.ieee.org/lpdocs/epic03/wrapper.htm?arnumber=6402423

[10] Mariani B, Hoskovec C, Rochat S, Büla C, Penders J, Aminian K. 3D gait assessment in young and elderly subjects using foot-worn inertial sensors. J Biomech. 2010;43: 2999–3006. doi: 10.1016/j.jbiomech.2010.07.003 20656291 10.1016/j.jbiomech.2010.07.003

[11] Mcginnis RS, Cain SM, Davidson SP, Vitali R V, Mclean SG, Perkins NC. Inertial sensor and cluster analysis for discriminating agility run Technique. Biomed Signal Process Control. 2017;32: 150–156. doi: 10.1016/j.ifacol.2015.10.177

[12] Chardonnens J, Favre J, Cuendet F, Gremion G, Aminian K. A system to measure the kinematics during the entire ski jump sequence using inertial sensors. J Biomech. 2013;46: 56–62. doi: 10.1016/j.jbiomech.2012.10.005 23123073 10.1016/j.jbiomech.2012.10.005

[13] Willmann RD, Lanfermann G, Saini P, Timmermans A, te Vrugt J, Winter S. Home stroke rehabilitation for the upper limbs. Conf Proc. Annu Int Conf IEEE Eng Med Biol Soc IEEE Eng Med Biol Soc Annu Conf. 2007;2007: 4015–8. doi: 10.1109/IEMBS.2007.4353214 18002880 10.1109/IEMBS.2007.4353214

[14] Sheppard JM, Young WB. Agility literature review: Classifications, training and testing. J Sports Sci. 2006;24: 919–932. doi: 10.1080/02640410500457109 16882626 10.1080/02640410500457109

[15] Zaferiou AM, Wilcox RR, McNitt-Gray JL. Modification of impulse generation during pirouette turns with increased rotational demands. J Appl Biomech. 2016;32: 425–432. http://dx.doi.org/10.1123/jab.2015-0314 27046934 10.1123/jab.2015-0314

[16] Zaferiou AM, Wilcox RR, McNitt-Gray JL. Modification of impulse generation during piqué turns with increased rotational demands. Hum Mov Sci. 2016;47: 220–230. http://dx.doi.org/10.1016/j.humov.2016.03.012 27038006 10.1016/j.humov.2016.03.012

[17] Young W, Farrow D. A review of agility: practical applications for strength and conditioning. Strength Cond J. 2006;28: 24–29. doi: 10.1519/00126548-200610000-00006

[18] Fino PC, Frames CW, Lockhart TE. Classifying step and spin turns using wireless gyroscopes and implications for fall risk assessments. Sensors (Basel). 2015;15: 10676–10685. doi: 10.3390/s150510676 25954950 10.3390/s150510676 PMC4481922

[19] Horak FB, Mancini M. Objective biomarkers of balance and gait for parkinson’s disease using body worn sensors. Mov Disord. 2013;28: 1544–1551. doi: 10.1002/mds.25684 24132842 10.1002/mds.25684 PMC3927718

[20] Novak D, Goršič M, Podobnik J, Munih M. Toward real-time automated detection of turns during gait using wearable inertial measurement units. Sensors (Basel). 2014;14: 18800–22. doi: 10.3390/s141018800 25310470 10.3390/s141018800 PMC4239865

[21] Pham MH, Elshehabi M, Haertner L, Heger T, Hobert MA, Faber GS, et al Algorithm for turning detection and analysis validated under home-like conditions in patients with parkinson’s disease and older adults using a 6 degree-of-freedom inertial measurement unit at the lower back. Front Neurol. 2017;8: 135 doi: 10.3389/fneur.2017.00135 28443059 10.3389/fneur.2017.00135 PMC5385627

[22] Yu G, Jang YJ, Kim J, Kim JH, Kim HY, Kim K, et al Potential of IMU sensors in performance analysis of professional alpine skiers. Sensors (Switzerland). 2016;16: 1–21. doi: 10.3390/s16040463 27043579 10.3390/s16040463 PMC4850977

[23] Mancini M, Schlueter H, El-Gohary M, Mattek N, Duncan C, Kaye J, et al Continuous monitoring of turning mobility and its association to falls and cognitive function: A pilot study. Journals Gerontol—Ser A Biol Sci Med Sci. 2016;71: 1102–1108. doi: 10.1093/gerona/glw019 26916339 10.1093/gerona/glw019 PMC5007616

[24] El-Gohary M, Pearson S, McNames J, Mancini M, Horak F, Mellone S, et al Continuous monitoring of turning in patients with movement disability. Sensors (Basel). 2013;14: 356–69. doi: 10.3390/s140100356 24379043 10.3390/s140100356 PMC3926561

[25] Ojeda L, Borenstein J. Non-GPS navigation for security personnel and first responders. J Navig. 2007;60: 391–407. doi: 10.1017/S0373463307004286

[26] Savage PG. Strapdown inertial navigation integration algorithm design Part 2: Velocity and position algorithms. J Guid Control Dyn. 1998;21: 208–221. doi: 10.2514/2.4242

[27] Cain SM, McGinnis RS, Davidson SP, Vitali R V., Perkins NC, McLean SG. Quantifying performance and effects of load carriage during a challenging balancing task using an array of wireless inertial sensors. Gait Posture. 2016;43: 65–69. doi: 10.1016/j.gaitpost.2015.10.022 26669954 10.1016/j.gaitpost.2015.10.022

[28] Orendurff MS, Segal AD, Klute GK, Berge JS, Rohr ES, Kadel NJ. The effect of walking speed on center of mass displacement. J Rehabil Res Dev. 2004;41: 829–834. doi: 10.1682/JRRD.2003.10.0150 15685471 10.1682/jrrd.2003.10.0150

[29] Wilcox R. Modern Statistics fo the Social and Behavioral Sciences. Boca Raton, FL: Taylor & Francis Group; 2012.

[30] Spörri J, Kröll J, Schwameder H, Müller E. Turn characteristics of a top world class athlete in giant slalom: A case study assessing current performance prediction concepts. Int J Sport Sci Coach. 2012;7: 647–660. doi: 10.1260/1747-9541.7.4.647

[31] Tan H, Wilson AM. Grip and limb force limits to turning performance in competition horses. Proc Biol Sci. 2011;278: 2105–11. doi: 10.1098/rspb.2010.2395 21147799 10.1098/rspb.2010.2395 PMC3107634

[32] Flash T, Hogan N. The coordination of arm movements: an experimentally confirmed mathematical model. J Neurosci. 1985;5: 1688–1703. 4020415 10.1523/JNEUROSCI.05-07-01688.1985 PMC6565116

[33] Thigpen MT, Light KE, Creel GL, Flynn SM. Turning difficulty characteristics of adults aged 65 years or older. Phys Ther. 2000;80: 1174–1187. 11087304

[34] Fuller JR, Adkin AL, Vallis LA. Strategies used by older adults to change travel direction. Gait Posture. 2007;25: 393–400. doi: 10.1016/j.gaitpost.2006.05.013 17064904 10.1016/j.gaitpost.2006.05.013

[35] Orendurff MS, Segal AD, Berge JS, Flick KC, Spanier D, Klute GK. The kinematics and kinetics of turning: limb asymmetries associated with walking a circular path. Gait Posture. 2006;23: 106–11. doi: 10.1016/j.gaitpost.2004.12.008 16311202 10.1016/j.gaitpost.2004.12.008

[36] Vitali RV, Cain SM, McGinnis RS, Zaferiou AM, Ojeda LV, Davidson S P, Perkins N C. Method for Estimating Three-Dimensional Knee Rotations Using Two Inertial Measurement Units: Validation with a Coordinate Measurement Machine. Sensors. 2017;17: 1970 doi: 10.3390/s17091970 28846613 10.3390/s17091970 PMC5620966

[37] Zaferiou AM, Flashner H, Wilcox RR, McNitt-Gray JL. Lower extremity control during turns initiated with and without hip external rotation. J Biomech. Elsevier; 2017;52: 130–139. doi: 10.1016/j.jbiomech.2016.12.017 28057348 10.1016/j.jbiomech.2016.12.017

[38] Wang H, Zheng N. Knee rotation and loading during spin and step turn. Int J Sports Med. 2010;31: 742–746. doi: 10.1055/s-0030-1261942 20645235 10.1055/s-0030-1261942

